# The effect of (-)-epigallocatechin gallate as an adjunct to non-surgical periodontal treatment: a randomized clinical trial

**DOI:** 10.1186/s13063-022-06298-6

**Published:** 2022-05-03

**Authors:** Jiajun Zeng, Yanfeng Wang, Qiao Yuan, Qingxian Luan

**Affiliations:** Department of Periodontology, Peking University School and Hospital of Stomatology, Beijing, People’s Republic of China

**Keywords:** Epigallocatechin gallate, Catechin, Ultrasound, Scaling and root planing, Periodontitis

## Abstract

**Background:**

EGCG is proven to be of good effect to relieve periodontal inflammation, but it has not been applied as a local delivery medicine in patients with periodontitis widely. The aim of this clinical trial was to evaluate the adjunctive effect of (-)-epigallocatechin gallate (EGCG) aqueous solution as a coolant during scaling and root planing in the management of chronic periodontitis.

**Methods:**

A double-blind, randomized controlled study was performed on 15 patients with moderate to severe chronic periodontitis. The bilateral maxillary teeth were randomly divided into the test side and the control side on every individual. On the control side, the periodontal therapy was routinely performed. And on the test side, in the process of periodontal therapy, the distilled water in the ultrasonic scaler was replaced with a 5-mg/mL EGCG solution. The probing depth (PPD), clinical attachment level (CAL), bleeding index (BI), gingival index (GI), and plaque index (PI) were recorded at baseline and 6 and 12 weeks after the treatment.

**Results:**

PPD, CAL, BI, GI, and PI generally improved after treatment in both groups. At the sixth week and the twelfth week of review, PPD, CAL, GI, and PI had no statistical difference (*p* >0.05) between the two groups. At the review of the twelfth week, BI on the test side decreased significantly (*p* <0.05).

**Conclusions:**

Using EGCG solution as the irrigant instead of water has an additional benefit on the bleeding index at the 12-week review. However, the rest clinical parameters had no additional benefit.

**Trial registration:**

ClinicalTrials.gov ChiCTR2000029831, date of registration: Feb 15, 2020.

**Supplementary Information:**

The online version contains supplementary material available at 10.1186/s13063-022-06298-6.

## Introduction

Periodontitis is defined as an inflammatory disease caused by specific groups of microorganisms and the host defense system [1]. The destructive results of periodontal inflammation include breakdown of alveolar bone, attachment loss of junctional epithelium and eventually loosening of teeth. The initial treatment for periodontitis is composed of daily using a dental brush and floss to control dental plaques, and receiving professional scaling and root planning to remove periodontal pathogens [2]. When dealing with the deep pockets or furcation involvements, however, clinical practitioners cannot always clean them firmly by conventional hand instruments and ultrasonic devices [3, 4]. Therefore, more and more researchers focus on generally or locally applying antimicrobial medicine as an adjunct to scaling and root planning, which proved beneficial to control periodontal infections [5–7]. Systemically using antimicrobial medicine, such as metronidazole and amoxicillin, delivers effective ingredients to the bottom of deep pockets and furcation of teeth through blood serum [8]. However, general application of antimicrobial medicine for a long term brings a lot of side effects which include allergy, drug resistance, fungal infections, and drug interactions. Compared with that, localized delivered drugs maintain a high concentration in gingival cervical fluids directly, which might reduce the occurrence of adverse reactions.

As the most abundant ingredient in green tea catechin, EGCG also has the highest biological activity which inhibits the growth of both gram-positive rods and gram-negative rods. The bacteriostatic abilities of EGCG could be explained that it damages bacterial cellular membrane that exerts an important influence when bacteria attach to host cells, while it reduces the activity of bacterial syntheses related to toxic products [9]. It was reported that the minimum inhibitory concentration of green tea catechin against *Porphyromonas gingivalis* and *Prevotella* spp. in vitro is 1.0 mg/ml [10]. At a concentration of 250-500 μg/mL, EGCG could restrain *P. gingivalis* to adhere to epithelial cells [11]. Sakanaka et al. [12] demonstrated that ECGC inhibits the synthetic process of n-Butyric acid and propionic acid in *P. gingivalis*. EGCG are also known inhibitors of cysteine proteinases of *P. gingivalis* and protein tyrosine phosphatase in *Prevotella intermedia* [13]. Therefore, EGCG has a potential to decrease periodontal damage from pathogens. In the meantime, EGCG regulates the host immune system to reduce inflammation. Once host cells get invaded by bacteria and their toxic products, a series of inflammatory factors will be released and trigger hydrolase as well as reactive oxygen species (ROS), which irritate osteoclasts to erode alveolar bone. Some researches has proved that EGCG acts as a cleaner for ROS in vivo. On the other hand, hydrolase, such as matrix metalloproteinase-1(MMP-1), MMP-8, and MMP-13, could accelerate collagen fibers’ collapse in periodontal tissue. But EGCG is demonstrated to inhibit the activity of MMP, which may contribute to maintain periodontal health [9, 14].

Recently, many researchers use green tea catechin as a local delivery medicine to treat periodontitis. Some of them applied hydroxypropyl cellulose (HPC) strips containing green tea catechin in deep pockets as a slow release medicine, and clinical improvements of patients’ periodontal status were achieved [10, 15, 16]. And some studies processed green tea catechin to gel form placed into pockets of patients with chronic periodontitis [17, 18]. The clinical and microbiological effects of the catechin were determined, which was beneficial to control periodontitis. However, the follow-up periods of the investigations above were within 8 weeks, and there lack long-time observation to prove the stable effects of green tea catechin in treating periodontitis.

EGCG is proven to be of good effect to relieve periodontal inflammation, but it has not been applied as a local delivery medicine in patients with periodontitis widely. For the reason, researches yet did not include a long-time follow-up beyond 3 months and there was a lot of heterogeneity about the results of these studies. In the present study, we applied EGCG solution (5mg/mL) as a water supply for the ultrasonic device during scaling and root planing. The additional beneficial effects of this new delivery system for EGCG as an adjunct to scaling and root planning were determined.

## Materials and methods

### Materials

(-)-Epigallocatechin gallate (EGCG, 94% purity) was purchased from Sunphenon EGCG® (Taiyo Green Power Company, Wuxi, China). The ultrasonic scaler was SKL A7 (frequency: 24–33kHz) purchased from SKL Medical Instrument Company (Guangdong, China).

The EGCG powder was dissolved in distilled water about half an hour before each clinical trial to prepare a 5-mg/mL aqueous solution of EGCG for use. It was colorless and transparent and identical in appearance with distilled water. The EGCG solution was filled in a bottle wrapped up with tinfoil and tightened the cap, in order to isolate air and light.

### High-performance liquid chromatography (HPLC) analysis

In order to detect the purity of EGCG powder, we used HPLC. Analytical conditions were referred our previous study [19]. Chromatographic analysis was performed on a Waters 2695 Series (Waters Technologies Shanghai Limited, Shanghai, China) LC system containing a quaternary pump, an online degasser, an autosampler, and a thermostatic column.

### Subject selection

The study protocol was documentarily approved by the Ethics Committee of Peking University School of Stomatology (No. PKUSSIRB-20183912) and was conducted in accordance with the Helsinki Declaration of 1975, as revised in 2000. It was registered in ClinicalTrials.gov (ChiCTR2000029831). Patients who were diagnosed as chronic periodontitis (with moderate-to-severe periodontitis according to the 1999 International Classification [20]) and referred for periodontitis treatment at the Department of Periodontics, Peking University School of Stomatology were invited to enroll in the study. The selected subjects were explained about the study and those who decided to enroll in the study had to sign the informed consent.

The inclusion criteria were as follows:

1. Age between 35 and 55 years old.
2. At least 2 teeth in each posterior region (upper right, lower right, upper left, lower left posterior) had a probing depth of 5–8 mm.

The exclusion criteria were as follows:

1. Patients having systemic diseases.
2. Patients who have received any topical or systemic antimicrobial treatment in the past 6 months, including the use of mouthwash.
3. Patients who had periodontal treatment in the past 6 months.
4. Pregnant and lactating mothers.
5. Less than 10 teeth remaining in the upper jaw except the third molar teeth.
6. Smokers.

### Clinical procedure

A split-mouth design was followed in the contralateral quadrants. This study was a double-blind, randomized, controlled clinical study. The upper jaw of the patient was randomly divided into the control side and test side according to a computer-generated list of random numbers by an investigator with no clinical involvement in the trial, and the investigator prepared a solution.

Two weeks before baseline, all the subjects signed the informed consent and received oral hygiene instruction and supra-gingival scaling. Two physicians were randomly divided as examiner and operator for one subject. The Kappa value of the consistency check between two physicians was 0.925, and the Kappa values of the consistency check with the chief physician were 0.925 and 0.935, respectively. Baseline probing depth, bleeding index, gingival index, plaque index, and clinical attachment level were recorded by the examiner. The operator performed the treatment and divided two sides into the control side and the test side. The examiner and operator of one subject were permanent. Teeth of test side received subgingival scaling with hand instrument and ultrasonic scaler that distilled water in which was replaced with 5 mg/mL EGCG solution. The teeth of the control side received normal subgingival scaling with a hand instrument and ultrasonic scaler. The time spent on each therapy was about 30 min.

**Fig. 1:**
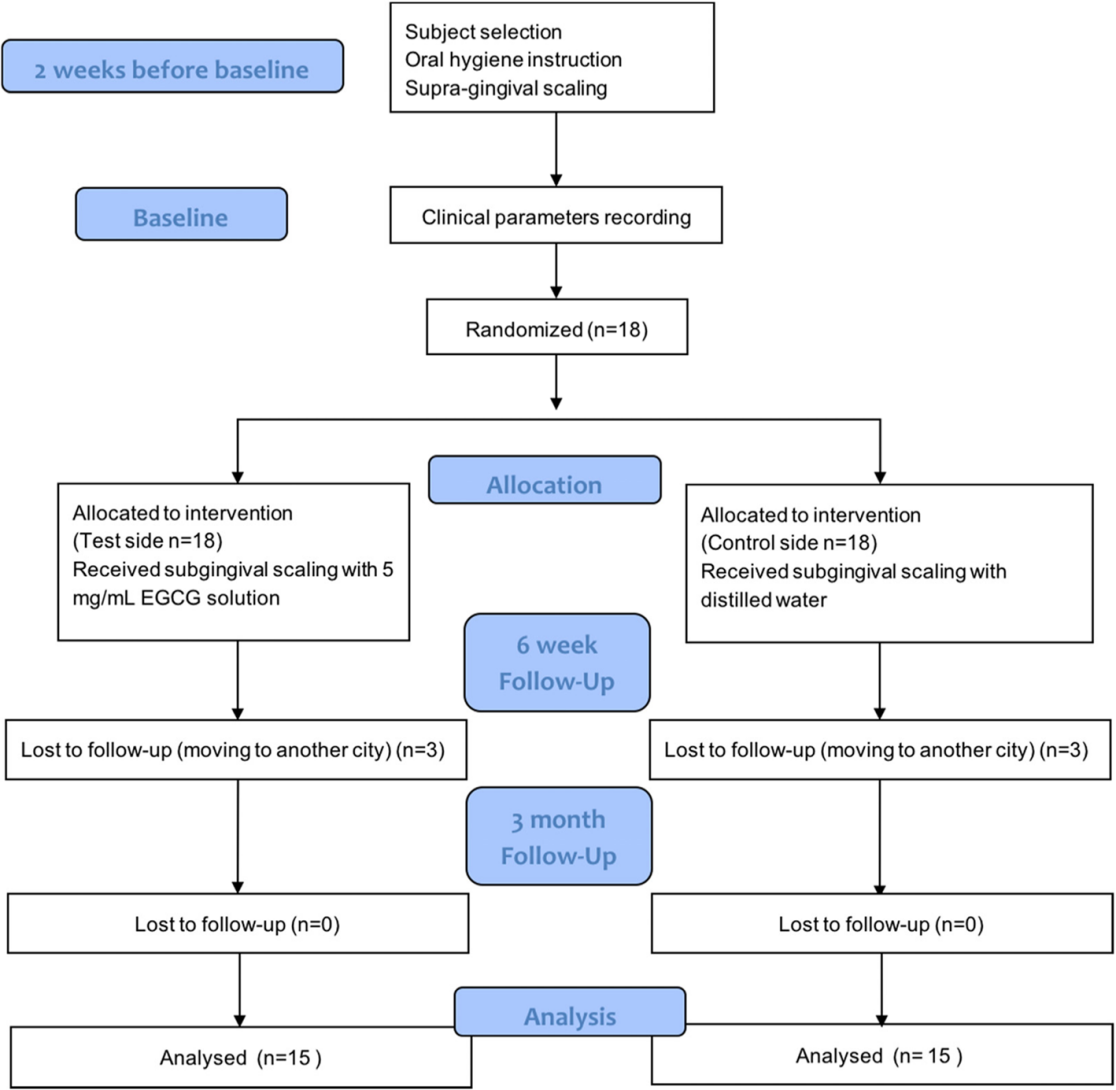
Flowchart of the study

All the recordings of clinical parameters were repeated at the end of 6th and 12th week after baseline (Fig. 1). At the review of the 6th week, two sides received ultrasonic scaling with EGCG solution and distilled water, respectively.

### Clinical parameters

GI (Gingival Index, Silness & Löe), PPD (Probing Pocket Depth), CAL (Clinical Attachment Level), PLI (Plaque Index, Silness & Löe), and BI (Bleeding Index, Mazza) were recorded using a periodontal probe (Hu-Friedy, Chicago, USA) at baseline and 6 weeks and 12 weeks. All the parameters were assessed at the test and control sites.

### Sample size calculation

Prior to the study, the sample size was calculated according to the standard deviation of the percent of bleeding on probing (BOP) obtained from a previous study [18]. At a 2-sided type I error of 0.05, 80% power of detection, a sample of 15 subjects was required. To compensate for potential dropouts, 18 subjects were recruited for the study.

### Statistical analyses

Statistical analysis was achieved by statistical software SPSS for Windows Version 22.0. Repeated measures analysis of variance test was used in the between-group comparison of slopes, and Multivariate analysis of variance was used at each time point. Paired-sample *t* test was used in the intra-group comparison, and a *p* value < 0.05 was accepted as significant.

## Results

Before the clinical trial, the purity of EGCG powder was detected. Table 1 showed that the purity of EGCG was 92–93%. Fifteen out of 18 subjects completed the study, and 3 subjects dropped out because of moving to another city. None of the subjects has complained or experienced adverse reactions after treatment. Table 2 showed the subject characteristics. There were 7 males and 8 females involved in the study, the mean age was 36.67 ± 6.70 years old. 612 and 600 sites were involved in the test side and control side respectively. At baseline, the mean PPD was 4.50 ± 1.57mm, mean CAL was 4.70 ± 2.17mm, mean BI was 3.23 ± 0.84, mean BOP was 99.01 ± 9.91%, mean PLI was 1.58 ± 0.68, mean GI was 2.48 ± 0.52.

**Table 1:** The purity of EGCG, detected by high performance liquid chromatography (HPLC) analysis

Prepared concentration (mg/mL)	Detected concentration (mg/mL)	Purity	Mean
1.0000	0.9326	93.26%	92.74 ± 0.74%
1.0000	0.9216	92.16%	
1.0000	0.9221	92.21%

**Table 2:** Demographic data at baseline

Characteristics	Data
Gender (male, female)	(7, 8)
Mean age (years)	36.67 ± 6.70
Number of sites	Test: 612; control: 600
Number of sites (PPD≥4mm)	Test: 412; control: 382
Number of sites (PPD≥6mm)	Test: 148; control: 126
Mean PPD	4.50 ± 1.57 mm
Mean CAL	4.70 ± 2.17 mm
Mean BI	3.23 ± 0.84
Mean BOP	99.01 ± 9.91%
Mean PLI	1.58 ± 0.68
Mean GI	2.48 ± 0.52

### Probing pocket depth and clinical attachment level (sites of PPD≥ 4mm at baseline)

At baseline, PPD was not significantly different between the test side and control side (5.30 ± 1.27mm vs 5.19 ± 1.23mm, *p=*0.181) (Table 3). At the 6th week and 12th week, PPD had statistical differences when compared with baseline in both sides (*p*<0.001). Mixed models were employed to account for the correlated data of PPD. However, comparison between groups revealed a similar change over time and showed an equal slope of quadratic mixed models. Both of the reduction was on the range of 1–1.3mm at the 6th week and of 1.7–2mm at the 3rd month, with no statistical difference between sides at any time point.

**Table 3:** Clinical variables (mean ± standard deviation) of probing pocket depth (PPD) and clinical attachment level (CAL) at baseline (T0), 6 weeks after baseline (T1), and 12 weeks after baseline (T2) (sites of PPD≥ 4mm at baseline)

		Mean *±* standard deviation			Between-group *p* value	Intra-group *p* value
		T0	T1	T2	Comparison of slopes	T0 vs T1 T0 vs T2
PPD (mm)	Test	5.30 *±* 1.27	4.04 *±* 1.35	3.57 *±* 1.30	*p* =0.680	*p* <0.001* *p* <0.001*
	Control	5.19 *±* 1.23	4.02 *±* 1.21	3.57 *±* 1.08		*p* <0.001* *p* <0.001*
Comparison 2 groups at time point		*p* =0.181	*p* =0.888	*p* =0.960		
CAL (mm)	Test	5.45 ± 2.02	4.41 ± 1.86	3.71 ± 2.01	*p* =0.918	*p* <0.001* *p* <0.001*
	Control	5.35 ± 1.89	4.45 ± 1.72	3.77 ± 1.87		*p* <0.001* *p* <0.001*
Comparison 2 groups at time point		*p* =0.448	*p* =0.798	*p* =0.672		

Repeated measures analysis of variance test was used in the between-group comparison of slopes. Multivariate analysis of variance was used in between-group comparison at each time point. Paired-sample *t* test was used in the intra-group comparison

*p <0.05

At baseline, CAL was not significantly different between the test side and control side (5.45 ± 2.02mm vs 5.35 ± 1.89mm). Equally, At the 6th week and 12th month, CAL had statistical differences when compared with baseline in both sides (*p*<0.001). However, comparison between groups also revealed a similar change over time.

### Probing pocket depth and clinical attachment level (sites of PPD≥ 6mm at baseline)

At baseline, PPD was not significantly different between the test side and control side (6.85 ± 0.91mm vs 6.83 ± 0.79mm, *p=*0.910) (Table 4). At the 6th week and 3rd month, PPD had statistical differences when compared with baseline on both sides (*p*<0.001). However, comparison between groups revealed a similar change over time and showed an equal slope of quadratic mixed models.

**Table 4:** Clinical variables (mean ± standard deviation) of probing pocket depth (PPD) and clinical attachment level (CAL) at baseline (T0), 6 weeks after baseline (T1), and 12 weeks after baseline (T2) (sites of PPD≥ 6mm at baseline)

		Mean *±* standard deviation			Between-group *p* value	Intra-group *p* value
		T0	T1	T2	Comparison of slopes	T0 vs T1 T0 vs T2
PPD (mm)	Test	6.85 *±* 0.91	5.04 *±* 1.55	4.38 *±* 1.58	*p* =0.658	*p* <0.001* *p* <0.001*
	Control	6.83 *±* 0.79	5.02 *±* 1.33	4.28 *±* 1.29		*p* <0.001* *p* <0.001*
Comparison 2 groups at time point		*p* =0.910	*p* =0.887	*p* =0.563		
CAL (mm)	Test	7.13 ± 1.89	5.59 ± 1.98	4.81 ± 2.11	*p* =0.806	*p* <0.001* *p* <0.001*
	Control	7.12 ± 1.47	5.69 ± 1.66	4.79 ± 1.71		*p* <0.001* *p* <0.001*
Comparison 2 groups at time point		*p* =0.934	*p* =0.973	*p* =0.914		

Repeated measures analysis of variance test was used in the between-group comparison of slopes. Multivariate analysis of variance was used in between-group comparison at each time point. Paired-sample *t* test was used in the intra-group comparison

*p <0.05

At baseline, CAL was not significantly different between test side and control side (7.13 ± 1.89mm vs 7.12 ± 1.47mm). Equally, At the 6th week and 12th week, CAL had statistical differences when compared with baseline on both sides (*p*<0.001). However, comparison between groups also revealed a similar change over time.

### Plaque index, gingival index, and bleeding index

At baseline, most of the selected sites exhibited moderate to severe gingival inflammation. PLI and GI were 1.60 ± 0.71 and 2.57 ± 0.50 at baseline, respectively, with no significant difference (Table 5). At the 6th week and 12th week, PLI and GI were significantly decreased in both sides when compared with baseline (*p*<0.001). However, PLI and GI on the two sides revealed a similar change over time, with no statistical difference between sides at any time point.

**Table 5:** Clinical variables (mean ± standard deviation) of plaque index (PLI), gingival index (GI), and bleeding index (BI) at baseline (T0), 6 weeks after baseline (T1), and 12 weeks after baseline (T2)

		Mean *±* standard deviation			Between-group *p* value	Intra-group *p* value
		T0	T1	T2	Comparison of slopes	T0 vs T1 T0 vs T2
PLI	Test	1.63 *±* 0.71	0.78 *±* 0.68	0.82 *±* 0.66	*p* =0.675	*p* <0.001* *p* <0.001*
	Control	1.57 *±* 0.71	0.79 *±* 0.62	0.82 *±* 0.66		*p* <0.001* *p* <0.001*
Between-group *p* value		*p* =0.232	*p* =0.761	*p* =0.964		
GI	Test	2.56 ± 0.50	1.86 ± 0.53	1.68 ± 0.55	*p* =0.219	*p* <0.001* *p* <0.001*
	Control	2.58 ± 0.49	1.88 ± 0.49	1.76 ± 0.54		*p* <0.001* *p* <0.001*
Between-group *p* value		*p* =0.559	*p* =0.568	*p* =0.539		
BI	Test	3.41*±* 0.73	2.12 ± 0.82	1.85 ± 0.76	*p* =0.219	*p* <0.001* *p* <0.001*
	Control	3.43*±* 0.74	2.10 ± 0.74	1.99 ± 0.80		*p* <0.001* *p* <0.001*
Between-group *p* value		*p* =0.733	*p* =0.723	*p* =0.014*		

Repeated measures analysis of variance test was used in the between-group comparison of slopes. Multivariate analysis of variance was used in between-group comparison at each time point. Paired-sample *t* test was used in the intra-group comparison

*p <0.05

As for BI, there was no statistical difference between the test and control sides at baseline (3.41 ± 0.73 vs 3.42 ± 0.74). At the 6th week and 12th week, BI had statistical differences when compared with baseline in both sides (*p*<0.001). When compared between groups, the mean reduction of BI of the test side was significantly higher than the control side at the 12^th^ month (*p*=0.014). However, comparison between groups revealed a similar change over time as shown by an equal slope of quadratic mixed models. On the other hand, we also calculated the BOP. At baseline, BOP of both sides were 100%, and comparison between groups revealed a similar change over time.

## Discussion

The main goal of the treatment of periodontitis is to remove the plaque formed by the subgingival flora on the root surface. Scaling and root planing is still the most effective methods for mechanically removing plaque. However, due to the complex anatomical shape of the teeth, the instrument cannot effectively reach the infected area, so it is difficult to completely remove the plaque [21–24]. In order to achieve better plaque clearance, many studies have combined topical antibiotics or antibacterials in periodontal non-surgical treatment [25–28]. It was reported that SRP combined with topical use of minocycline could reduce the probing depth by an additional 0.49 mm, and topical use of tetracycline can reduce the probing depth by an additional 0.47 mm. Using metronidazole and chlorhexidine could also get similar results. However, because the elution rate of the drug in the periodontal pocket was very fast and the maintenance time was short, a higher concentration was needed to increase the bacteriostatic effect and maintain a longer time, which may cause some controversy [29–31], such as antibiotics may cause bacterial resistance [32]. As an alternative to antibiotics, chlorhexidine had a low possibility for inducing bacterial resistance. However, due to the damaging effect of chlorhexidine on the protein, when chlorine was transported into the periodontal pocket during periodontal treatment, it may be detrimental to periodontal tissue healing [33].

As a natural product, EGCG has been widely recognized for its ability to resist inflammation and anti-oxidation and inhibit *P. gingivalis* and *Plasmodium* [13, 34]. In addition to resisting inflammation and inhibiting microorganisms, EGCG can also inhibit MMP-9 expression in osteoblasts, reduce osteoclast formation, and induce osteoclast apoptosis to reduce alveolar bone absorption through caspase-mediated apoptosis [13, 34]. It was also demonstrated in animal experiments that it inhibited the absorption of alveolar bone. EGCG can inhibit inflammatory alveolar bone resorption induced by lipopolysaccharide by inhibiting membrane-bound prostaglandin synthetase 1 and 2 (mPGES-1, mPGES-2) expression, thereby reducing prostaglandin E2 induced by lipopolysaccharide. At the same time, EGCG can also inhibit RANKL and prevent osteoblasts from differentiating into osteoclasts [35]. In addition, in recent years, studies have been conducted on oral EGCG in mice with periodontitis, attachment loss, alveolar bone resorption, and expression of inflammatory factors were inhibited [36, 37]. Moreover, there are no reports of side effects in current clinical studies, so there is great potential for application in the treatment of periodontitis.

In this study, we used an ultrasonic scaler as a means of delivering drugs that differed from previous studies. The premise of using this method is that the EGCG solution did not significantly decrease after being atomized through the ultrasonic scaler, and our previous study has shown that the 2 mg/mL EGCG solution was stable, and 5 mg/mL was used in this clinical study, which was more stable [19].In terms of concentration selection, studies by Asah et al. [38] and Hiraasaw et al. [10] showed that 0.50 mg/mL and 1.00 mg/mL were the lowest EGCG inhibitory concentrations, respectively, whereas gel or chips were used in the past, and the concentration previous studies used was mostly above 10 mg/mL [17, 18], so the concentration used in this study was safe and effective, and no patients indicated that there was any discomfort throughout the study. The experimental design method of split-mouth was because the inflammation of both sides of the chronic periodontitis patients was relatively similar without obvious local promotion factors. In this way, the effects of individual factors on the test side and the control side could be minimized.

In previous studies, Hiraasaw et al. [10] reported that at week 8, the mean probing depth in the test group which used catechin HPC chips was significantly lower than that of the control group which use placebo, but only 8 subjects were included. Kudva et al. [15] and Hattarki et al. [16] also used catechin HPC chips, studies showed probing depth, gingival index, and plaque index significantly improved at 21 days and 5 weeks. Chava et al. [17] delivered catechin in a thermoreversible slow-release gel into the periodontal pocket of patients with chronic periodontitis. After 4 weeks, probing depth, clinical attachment level, and the gingival index had significant improvement compared to the placebo-treated group. However, the longest-running study of Rattanasuwan et al. [18], which injected a gel of catechin into periodontal pockets, showed that from 1 to 6 months, probing depth, clinical attachment level, plaque index, or gingival index were not significantly different from those of the placebo-treated group. Only bleeding on probing had a statistical difference between the two groups at 3 months. Probing depth and clinical attachment level in our study also had no significant difference between the test side and control side, which was inconsistent with the previous research results. However, the improvement of the test side was larger than the control side, which seemed to show the potential effect. On the other hand, the bleeding index had significant improvement at 12 weeks. The reasons for the differences in the results may be that the frequency, concentration, and method of using EGCG differed from previous literature. In previous studies, whether using chips or a gelatinous agent, most of the repeated administrations were performed several times after subgingival scaling and root planing. But our study was only administered at baseline and was not repeated until the review, so the frequency of EGCG was lower than in the previous literature. In terms of concentration selection, as discussed above, the concentration of EGCG used in this study was lower than in previous studies and may have an impact on the results of the study.

As for the method of using EGCG, Gregory et al. [39] evaluated the penetration depth of the water coolant for medicament lavage of an ultrasonic scaler into periodontal pockets, using a stain instead of distilled water to eject from the tip of the ultrasonic scaler. The results showed that the probing depth from 3.0 to 9.0 mm, the stain could reach the position where the ultrasonic tip could reach, which had a linear relationship with the probing depth. But the disadvantage was that the area where the rinsing liquid reached was relatively limited, basically along the working path of the ultrasonic working tip, the dispersion of the dye-colored stain was localized to the area of the ultrasonic probe with very little lateral dispersion. The study implied that the ultrasonic instrument may be effective to mechanically remove plaque and calculus at the same time as delivering a chemotherapeutic agent to the base of the periodontal pocket. On the other hand, the teeth in the study of Gregory et al. [39] were clinically needed to be extracted, the inflammation of the gingiva was heavy, and the periodontal pockets were slack, probably were the reasons why the stain could reach 9.0 mm. We thought that the level of gingival inflammation, the tightness of the periodontal pockets would influence the penetration depth of periodontal pockets that the drug could reach in this method of administration. Moreover, Chapple et al. [40] and Taggart et al. [41] also used an ultrasonic scaler to deliver 0.02% chlorhexidine as an adjunct to scaling and root planing. And there was no statistically significant difference between the two groups. In conclusion, previous studies using tablets or gels for local administration may maintain a longer elution time in the periodontal pockets, whereas the present study used an ultrasound tip to deliver EGCG which had a short elution time. Comparing with the research of Wang et al. [42], a new-type scaler tip with the terminal outlet was applied, which could deliver EGCG solution to the bottom of periodontal pockets, the results showed a significant PPD reduction after 6 months. In this study, the solution originated from the base of the tip rather than the top of the tip, so the EGCG aqueous solution may only reach a limited area in periodontal pockets. Perhaps this was one of the reasons why EGCG in the present study did not play a significant role. On the other hand, the effect of EGCG may be covered up by scaling and root planing. For the periodontal pocket where the probing depth was not so deep, it would be improved by complete scaling and root planing, thus whether the use of EGCG could not be well-reflected.

In addition, the experimental design of the split-mouth could eliminate the individual differences between the test group and the control group as much as possible, but the accompanying problem was that the EGCG solution may flow to the control side during the treatment on the test side, which was an interference with the results of the control group. In order to avoid interference caused by these factors as much as possible, only maxillary teeth were chosen, and a strong suction was used in the course of treatment to further prevent the rinsing liquid from flowing to the opposite side.

In view of the above-mentioned problems, the subsequent research should include more deep periodontal pockets, on the one hand, and improve the drug-delivery method of EGCG, on the other hand, explore the appropriate concentration of EGCG solution, appropriately enlarge the sample size, and extend the observation time.

## Conclusions

Although the anti-inflammatory and microbial inhibitory effects of EGCG have been widely recognized, clinical studies on periodontitis have been rarely used, and the observation time was generally short, resulting in a heterogeneous result. In the present study, the ultrasonic work tip was used, and the EGCG aqueous solution was given as an adjunctive modality to scaling and root planing. The bleeding index had significant improvement at 12 weeks, while probing depth and clinical attachment level had no additional benefit, probably because of a short duration of EGCG, a low concentration of EGCG, or the method of drug delivery. Nevertheless, the results of the present study suggested that there were some benefits on EGCG for chronic periodontitis, and more researches are needed.

## Supplementary Information

**Additional file 1.**

## Data Availability

The following information was supplied regarding data availability: The raw data has been supplied as Supplemental Dataset Files.
